# The Role of PPAR Alpha in the Modulation of Innate Immunity

**DOI:** 10.3390/ijms221910545

**Published:** 2021-09-29

**Authors:** Maja Grabacka, Małgorzata Pierzchalska, Przemysław M. Płonka, Piotr Pierzchalski

**Affiliations:** 1 Department of Biotechnology and General Technology of Foods, Faculty of Food Technology, University of Agriculture, ul. Balicka 122, 30-149 Cracow, Poland; malgorzata.pierzchalska@urk.edu.pl; 2 Department of Biophysics, Faculty of Biochemistry, Biophysics and Biotechnology, Jagiellonian University, ul. Gronostajowa 7, 30-387 Cracow, Poland; przemyslaw.plonka@uj.edu.pl; 3 Department of Medical Physiology, Faculty of Health Sciences, Jagiellonian University Medical College, ul. Michałowskiego 12, 31-126 Cracow, Poland; piotr.pierzchalski@uj.edu.pl

**Keywords:** pattern-recognition receptors, phagocytosis, nitric oxide synthase, fenofibrate, oleoylethanolamide, palmitoylethanolamide

## Abstract

Peroxisome proliferator-activated receptor α is a potent regulator of systemic and cellular metabolism and energy homeostasis, but it also suppresses various inflammatory reactions. In this review, we focus on its role in the regulation of innate immunity; in particular, we discuss the PPARα interplay with inflammatory transcription factor signaling, pattern-recognition receptor signaling, and the endocannabinoid system. We also present examples of the PPARα-specific immunomodulatory functions during parasitic, bacterial, and viral infections, as well as approach several issues associated with innate immunity processes, such as the production of reactive nitrogen and oxygen species, phagocytosis, and the effector functions of macrophages, innate lymphoid cells, and mast cells. The described phenomena encourage the application of endogenous and pharmacological PPARα agonists to alleviate the disorders of immunological background and the development of new solutions that engage PPARα activation or suppression.

## 1. Introduction

Innate immunity comprises a sophisticated set of defensive processes, which are evolutionarily very old and originated concomitantly with the development of multicellular organisms. The defense against invading pathogens is a crucial physiological mechanism that guarantees survival. The development of these mechanisms is a manifestation of a constant race between pathogens (including unicellular pro- and eukaryotic invaders) and host. The biological processes involved in the innate immune response are very complex and tightly regulated on multiple levels, because they may be very harmful when left unsupervised. Recent advances in the elucidation of such a regulation revealed a dense network of connections among immune cell functions, signaling pathways, and cellular metabolism. Peroxisome proliferator-activated receptor α (PPARα) has emerged as an important player in this network, and this review aims to present several aspects of its involvement in the regulation of innate immunity.

## 2. The New Perspective on Innate Immunity

Innate immunity has evolved to react very rapidly to injury or invasion, and it involves an immediate mobilization of a broad range of inflammatory responses of rather low specificity. Traditionally, the lack of memory was regarded as an intrinsic feature of innate immunity; nevertheless, recent discoveries in this field have led to a thorough revision of this picture and a presentation of the concept of ‘innate immune memory’ (reviewed in [1]). The innate immune memory differs substantially from its adaptive counterpart, because it lacks somatic gene rearrangement processes and specific epitope-recognizing receptors. Due to the gradual improvement depending on the history of host–pathogen interactions, it is also called ‘trained immunity’, with genetic recombination events being substituted by the development of epigenetic imprinting and/or changes in miRNA transcriptome. The observations of the innate immune cells’ behavior during the exposure to various unrelated pathogens revealed the ‘priming’ phenomenon, whereby previous contact with one microbial component modulates the response to other pathogenic challenges [1,2]. This modulation can form a certain kind of cross-protection, which is manifested by a nonspecific improved resistance to second infection after an episode of pathogen-associated molecular pattern (PAMP) recognition by pattern-recognition receptors (PRRs) [2]. Such phenomena have been reported in insects (*Tenebrio mollitor* larvae) [3], in planaria (*Schmitdtea mediterranea*) [4], and in Pacific oyster *Crassostrea gigas* [5]. Notably, invertebrates, which lack lymphocyte-based adaptive immunity mechanisms and rely solely on innate responses to fight infections, have developed a high level of sequence diversity and structural complexity of PRRs (e.g., lectins, Toll-like receptors (TLRs), and NOD/NLR-like proteins (see Section 4.4)), as well as soluble or extracellular fibrinogen-related proteins (FREPs) [6,7]. Recognition of PAMPs, such as β-1,3-glucans and peptidoglycan, triggers specific invertebrate antimicrobial effector mechanisms, for instance, activation of pro-phenoloxidase (and related hemocyanins) that catalyze melanin formation from reactive dihydroxyphenylalanine (DOPA) and DOPAquinone intermediates [8,9].

The three main steps of the innate response are (1) building of a physical and chemical barrier, (2) recognition of foreign invaders and distinguishing from ‘self’ structural elements, and (3) phagocytosis and production of cytotoxic compounds that help to destroy engulfed particles or are released to damage objects too large to be phagocytosed. For example, various epithelial cells not only form a physical barrier of epithelium protecting the body from the external environment but also secrete hydrolytic enzymes and alarmins such as various antimicrobial peptides (AMPs) [10]. To distinguish between self and foreign molecules and cells, PRRs bind particular molecules characteristic for certain groups of common pathogens of viral, bacterial, or fungal origin, such as nucleic acids and their components (e.g., double-stranded RNA, nonmethylated CpG contacting DNA, nucleotides, and nucleosides), saccharide cell-wall components (e.g., peptidoglycan, lipopolysaccharide, chitin, and zymosan), phospholipids (i.e., cardiolipin of microbial origin), or particular proteins (e.g., formylmethionine-containing peptides and flagellin), usually regarded as PAMPs. The same mechanisms are responsible for the response to disrupted cell contents released during necrosis, which are immunogenic, such as mitochondrial formylated peptides, cardiolipin-containing inner mitochondrial membrane, and ATP (damage-associated molecular patterns, DAMPs) [11,12]. In a localization where invasion or sterile injury take place, phagocytosis leading to the elimination of a danger is triggered. It is carried out by professional phagocytes (polymorphonuclear neutrophils, mononuclear monocytes, and macrophages residing in tissues), para-professional phagocytes (dendritic cells), and nonprofessional phagocytes (epithelial cells and fibroblasts) [13,14].

During phagocytosis, the engulfed particles or microbial cells need to be destroyed intracellularly by a variety of microbicidal molecules stored in cytoplasmic granules, such as antimicrobial peptides (AMPs, e.g., azurocidin and defensins), proteolytic enzymes (e.g., elastase, cathepsin G, collagenase, gelatinase, and metalloproteinases), and reactive oxygen, nitrogen, and halogenated species [15]. Cytotoxic reactive oxygen species are generated during respiratory burst and include the superoxide anion (O^•−^), produced by NADPH oxidase, as well as hydrogen peroxide generated by superoxide dismutase from O^•−^. NADPH oxidase, which is assembled from the transmembrane cytochrome b558, numerous cytosolic phox (phagocyte oxidase) subunits, and small GTPase Rac2, releases O^•−^ directly into the phagosome or the extracellular space [16]. A small fraction of superoxide (about 1%) may give rise to a highly reactive hydroxyl radical in reaction with ferric ions (Fe^3+^) [16,17]. Neutrophil myeloperoxidase uses hydrogen peroxide and halides to form hypochlorous or hypobromous acids, as well as highly bactericidal chloramines. Mononuclear phagocytes express inducible nitric oxide synthase and produce cytotoxic nitric oxide (NO) from arginine. During the active phase of oxidative burst, NO, which freely diffuses across membranes, reacts with O^•−^, giving rise to peroxynitrite (ONOO^−^), a strong oxidative agent able to induce nitrative or oxidative damage to proteins and lipids of microbial cells [18]. At later stages of phagocytosis, the phagosome fuses with strongly acidic lysosomes to form phagolysosomes which also contain numerous hydrolytic enzymes, such as proteinases, lipases, and lysozyme.

## 3. The Main Populations of Innate Immune Cells

Professional phagocytes, such as neutrophils, monocytes/macrophages, or microglia, play a central role in innate immunity, because they perform both regulatory and effector tasks. Macrophages of peripheral tissues belong to the reticuloendothelial system and are known under various customary names according to localization: Kupffer cells (liver), Langerhans cells (skin), osteoclast (bone), etc. Microglia are also skilled phagocytes of myeloid origin that reside exclusively in the central nervous system and share numerous common features with macrophages [19]. The phagocytic capacity of monocytes and monocyte-derived macrophages depends on the expression pattern of specific surface markers, as well as their phenotypic polarization. A recent report [20] showed that M2 macrophages (stimulated with IL-4 and IL-10) presented a twofold higher phagocytic capacity of *E. coli* than M1 macrophages (IFNγ, LPS-stimulated), and the expression level of a surface marker CD209 directly correlated with a high phagocytic capacity. The plethora of stimuli determine which pathway the cell follows, called ‘polarization’. M1-polarized macrophages respond to so-called ‘classical’ activation by typical proinflammatory cytokines, such as IFNγ, secrete other proinflammatory factors (TNFα, IL-1β, IL-6, and IL-12) and chemokines (e.g., CCL1, CCL5, and CXCL10) to recruit other leukocyte populations, and release cytotoxic NO (see below). M2 macrophages represent an opposite, anti-inflammatory phenotype as a result of the so-called ‘alternative’ activation by IL-4, IL-13, parasitic (helminth, fungal) infections, or immunosuppressing factors, such as IL-10 and glucocorticoids. They express mannose receptor (CD206) and arginase-1, and they secrete the anti-inflammatory IL-10 cytokine, TGF-β, and trophic polyamines (putrescine, spermidine, etc.), collectively contributing to inflammation resolution and tissue regeneration [21,22]. The M1/M2 paradigm was recently broadened and enriched with further details, such as division of the M2 group into more specific M2a, M2b, M2c, and M2d phenotypes [23,24]. However, an opinion currently prevails that, due to macrophage plasticity, there is rather a continuum of phenotypes than distinct, exclusive, and restricted cell profiles [25].

In addition to the aforementioned professional and nonprofessional phagocytes, other cell populations take part in the innate immune defense, namely, innate lymphoid cells (ILCs) from lymphoid lineage and mast cells, eosinophils, basophils, and myeloid-derived suppressor cells from myeloid lineage [25]. Mast cells, secreting heparin and histamine, reside in many tissues and organs, such as connective tissue, skin, lungs, gastrointestinal mucosa, and in proximity to blood vessels [26].

Myeloid-derived suppressor cells (MDSCs) form a heterogenous and plastic population of cells of myeloid origin that inhibit T-cell responses and are able to promote differentiation toward Tregs [25,27]; therefore, they actively contribute to inflammation resolution by being recruited to the site of inflammation by proinflammatory cytokines, such as IL-6.

The last, most recently discovered, and somewhat elusive group of innate immunity effectors comprises so-called innate lymphoid cells (ILCs) [25,28]. They show a common pattern of surface markers (CD45^+^ CD127^+^ CD3^−^ CD19^−^) and are divided into three main groups (ILC1, ILC2, and ILC3) on the basis of the expression of particular transcription factors and a distinct profile of secreted cytokines [28,29,30]. Natural killer (NK) cells and large granular lymphocytes (LGLs) belong to ILC1 [31,32], whereas ILC2 and ILC3 cells are mainly associated with mucosal membranes [29,33]. ILC3 cells derived from fetal liver are among the first lymphoid cells that populate gastrointestinal tract, and they play an important role in the development of tolerance to commensal microbiota [34,35]. They secrete IL-17, IL-22, and lymphoid tissue inducer (LTi), which are critical factors for maintaining mucosal barrier function, sustaining the balance between the inflammatory response to pathogenic microbes, and creating the tolerogenic milieu for probiotic bacteria [28,35]. Collectively, ILC cells are involved in the coordination of various aspects of innate immunity and contribute to immune homeostasis regulation; therefore, they are regarded as an equivalent of Th lymphocytes in adaptive immunity.

## 4. Peroxisome Proliferator-Activated Receptor alpha (PPARα) and Its Role in Inflammation

**Figure 1:**
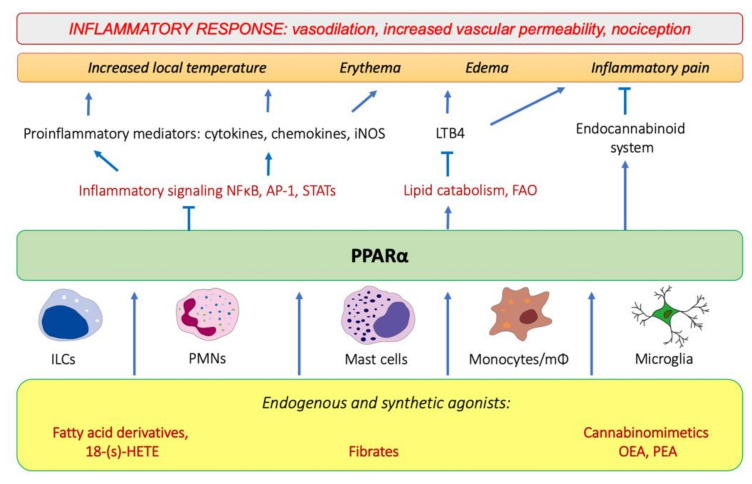
The involvement of PPARα in the modulation of inflammation through interfering with the main inflammatory transcription factors (NF-κB, nuclear factor κB; AP-1, activation protein 1; STATs, signal transducers and activators of transcription) through activating lipid catabolic pathways and participating in the endocannabinoid system (see Section 7). iNOS, inducible nitric oxide synthase; FAO, fatty-acid oxidation; LTB_4_, leukotriene B_4_; OEA, oleylethanolamide; PEA, palmitoylethanolamide.

Tissue injury and the onset of infection immediately evoke an innate immune response and trigger inflammation. As pointed out by Roman scholar Aulus Cornelius Celsus in the first century, local acute inflammation is manifested by *calor*, *rubor*, *dolor*, and *tumor*, i.e., increased temperature, redness, pain, and edema [36]. These symptoms reflect the action of proinflammatory lipid mediators, histamine, and cytokines released by tissue-infiltrating leukocytes that induce vasodilation and increase endothelial permeability and expression of adhesion molecules on the endothelial surface and in the extracellular matrix underneath. These events lead to extravasation of circulation leukocytes, chemotaxis, and accumulation of interstitial fluid, causing edema (*tumor*). The increased interstitial flow and metabolic activity of proliferating cells generate local heat and flushing (*calor* and *rubor*). Inflammatory pain (*dolor*) is evoked by activation of transient receptor potential cation channel vanilloid subfamily member 1 (TRPV1), which is present on sensory neurons of the peripheral nervous system [37]. The TRPV1 activation leads to an influx of Ca^2+^ and membrane depolarization, followed by the opening of voltage-gated sodium channels and creation of an action potential [37]. TRPV1 receptors are present not only on neurons, but also on immunocompetent cells (T lymphocytes, mast cells), epithelia, keratinocytes, and vascular endothelial cells [38]. TRPV1 channels are activated by various lipid inflammatory mediators, such as COX-2 products (prostaglandins), lipoxygenase 15-LOX products (e.g., 15-hydroperoxyeicosatetraenoic acid, 15-HPETE), and polyamines of molecules released after cell injury, e.g., ATP and adenosine [37]. The links between PPARα and molecular events that spark inflammation and underlie its main symptoms are outlined below (Figure 1).

### 4.1. PPARα as a Nuclear Receptor Present in Peripheral Tissues and Immunocompetent Cells

Peroxisome proliferator-activated receptors (PPARs) belong to a family of nuclear receptors that act as transcription factors activated by lipid-soluble ligands. Such ligands are able to cross the plasma membrane directly and bind the intracellular target proteins. PPARs are represented by three isotypes, PPARα, PPARβ/δ, and PPARγ, encoded by separate genes. They show tissue-specific expression patterns and mainly govern lipid, carbohydrate, and amino-acid metabolism, as well as exert other pleiotropic functions, including immunomodulatory activities. All three PPAR isotypes exhibit potent anti-inflammatory properties and have a strong impact on various aspects of the physiology of the immune system. In this review, we focus on peroxisome proliferator-activated receptor alpha (PPARα), which is particularly responsible for the regulation of fatty-acid catabolism and ketogenesis [39,40], also in addition to being deeply involved in the modulation of innate immunity responses. Below, we outline the active participation of PPARα in physiological processes that operate behind all four cardinal symptoms of inflammation, i.e., alleviating edema and pain and contributing to resolution of acute phase.

As a transcription factor, PPARα is involved in the activation of gene transcription, which is carried out by binding the heterodimer of PPARα and the pan-PPAR obligatory partner, retinoid X receptor (RXR), to consensus motifs in the target promoters. The active heterodimer is formed when both partners have their agonists bound. The most potent endogenous PPARα agonists include fatty acids and their derivatives: saturated stearic and palmitic acids, fatty acyl amides such as oleylethanolamide (OEA) and palmitoylethanolamide (PEA), LOX products such as 5-(*S*)-HETE and 8-(*S*)-HETE, and leukotriene B_4_ (LTB_4_) [41,42,43,44]. There is the only one bona fide RXR ligand known so far, which is 9-*cis*-13,14-dihydroretinoic acid, successfully identified after many years of searching, whereas 9-*cis*-retinoic acid, frequently used experimentally, is one of the most potent pharmacological RXR agonists [45,46]. Pharmacological PPARα agonists, such as fibrates, are clinically used to normalize blood lipid profile, particularly to lower concentrations of cholesterol and low-density lipoprotein fractions [47]. Fenofibrate and gemfibrozil are the most widely prescribed drugs from a fibrate group, and they are generally very well tolerated [48]. Nevertheless, some adverse effects have been reported in patients chronically taking fibrates, with myopathy and rhabdomyolysis being the most common problems [49]. The structures of endogenous ligands, as well as the most important synthetic agonists and antagonists, are presented in Table 1.

Interestingly, in addition to the tissues with a high rate of fatty-acid catabolism, such as the liver, cardiac muscle, and kidneys, PPARα is generally expressed in CD45^+^ leukocytes [50], including numerous innate immune cell populations: basophils [51], eosinophils [52], monocytes and macrophages [30,53,54,55], Kupffer cells [56], Langerhans cells [57], osteoclasts [58], and microglia [59].

The classical PPARα targets include the genes encoding enzymes from the fatty-acid mitochondrial and peroxisomal β-oxidation (acyl-CoA dehydrogenases, acyl-CoA oxidases), ω-oxidation and ω-hydroxylation (cytochromes P450), and ketogenesis (3-hydroxy-3methylglutaryl-CoA synthase) [60,61,62]. Importantly, in addition to this canonical mode of action, PPARα is able to transrepress certain genes through at least three mechanisms [63]: (i) initiating protein–protein interactions and sequestration of coactivators that are common to PPARα and other pathways, (ii) cross-coupling of the PPARα/RXR complex with other transcription factors, which leads to mutual cross-inhibition of both participating proteins, and (iii) interference with signal-transducing proteins, i.e., where the PPARα/RXR complex inhibits phosphorylation of MAP-kinase cascade members.

### 4.2. PPARα-Mediated Transrepression of Main Inflammatory Transcription Factors

**Figure 2:**
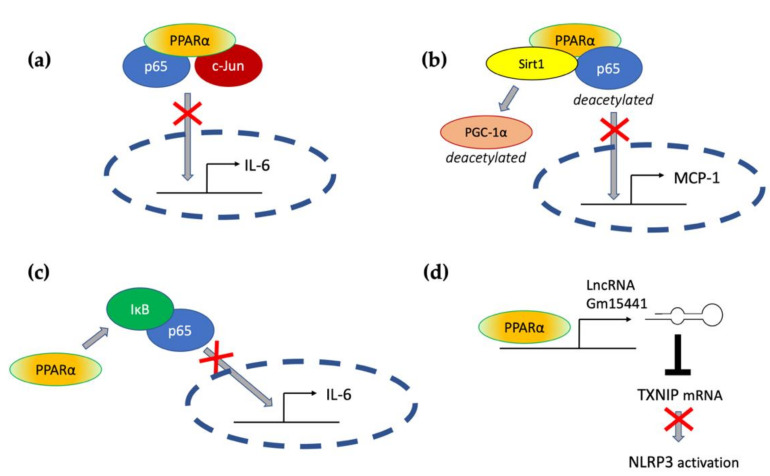
The molecular mechanisms responsible for PPARα-mediated suppression of proinflammatory signaling pathways (see the main text for explanation) (**a**) through a direct interaction with p65 and c-Jun, (**b**) through interaction with Sirt1 and subsequent deacetylation of p65, (**c**) through activation of IκB, and (**d**) through transactivation of long noncoding RNA Gm15441, which interferes with the stability of thioredoxin-interacting protein (TXNIP) mRNA and blocks NLRP3 inflammasome activation.

Transrepressive activity toward nuclear factor κB (NF-κB), activation protein (AP-1), and signal transducers and activators of transcription (STATs) is responsible for PPARα’s profound anti-inflammatory action. PPARα physically interacts with the p65 Rel homology domain through its C-terminal fragment and simultaneously binds the JNK-responsive part of c-Jun with its N-terminal fragment (Figure 2a) [65]. Formation of this complex sequesters p65 and c-Jun from binding to the IL-6 promoter and blocks IL-1-induced IL-6 production. The direct inhibitory interaction between PPARα and NF-κB p65 subunit was also reported in cardiomyocytes [66]. In this case, sirtuin 1 (Sirt1) initiated formation of the Sirt1–PPARα–p65 complex, which led to PPARα-dependent p65 inactivation and transrepression of proinflammatory NF-κB-regulated genes, such as monocyte chemoattractant protein 1 (MCP1, Figure 2b) [66]. Sirt1 induced p65 deacetylation, which also had a negative impact on NF-κB activity because acetylation is required for its activity [67]. The deacetylation effect was absent after treatment with PPARα antagonist GW6471 or in PPARα^−/−^ cells, which indicates PPARα involvement [66].

An additional mechanism responsible for PPARα interference with the NF-κB pathway was also identified in hepatocytes, where PPARα bound and transactivated NF-κB inhibitor alpha (IκBα), which increased the amount of this protein [68]. Accumulated IκBα binds NF-κB, thereby masking its nuclear localization signal, which arrests it in the cytoplasm and blocks its activity as a transcription factor [69]. PPARα was also responsible for the decreased phosphorylation of NF-κB subunits p65 and p50 [68], which was another event with a negative impact on NF-κB activity, because phosphorylation of its subunits is necessary for their optimal function [70]. The interference of PPARα with NF-κΒ action prevented IL-1 induced IL-6 expression in liver tissues (Figure 2c) [68].

The antagonism between PPARα and NF-κB and AP-1 underlies blocking of the expression of proinflammatory cytokines and effector proteins in various cell and animal models. PPARα ligand K-111 (2,2-dichloro-12-(4-chlorophenyl)-dodecanoic acid) inhibited LPS-induced IL-6 production in Raw 264.7 macrophages on the mRNA and protein level [71]. This effect was exerted through the inhibition of stress-activated protein kinase (SAPK)/c-Jun N-terminal kinase (JNK), NF-κB p65 phosphorylation, and induction of IκBα protein level [71]. PPARα activation in monocytes was shown to inhibit LPS- or IL-1β-induced expression of tissue factor (TF), a membrane glycoprotein responsible for initiation of coagulation cascade [72,73]. The mechanism involved a previously mentioned blockade of the target gene promoter activity through the antagonism between PPARα and NFκB and AP-1 [72].

Interleukins released by immune cells exert their biological functions through specific cell surface receptors, which transduce signals through the Janus family of kinases (JAK) and phosphorylation STAT transcription factors [74]. Various STAT proteins are negatively regulated by PPARα. For example, a bidirectional cross-inhibitory relationship between PPARα and STAT5b was described [75,76,77]. STAT5b is responsible for signal transduction from the IL-2 receptor [78]. IL-2 is a very important cytokine, crucial for both innate and adaptive immunity, being indispensable for NK cell proliferation and maturation, as well as promoting the development, differentiation, and proinflammatory response of both Th1 and Th2 cells [78,79].

### 4.3. PPARα and Inflammatory Lipid Mediators

Another important mechanism of the anti-inflammatory action of PPARα involves the catabolism of lipid mediators, such as leukotriene B_4_ (LTB_4_). The elegant study by Devchand and colleagues [80] revealed that LTB_4_ is a potent and specific PPARα ligand that induces expression of PPARα-transactivated genes of the peroxisomal β-oxidation pathway, namely, acyl-CoA oxidase, which is a rate-limiting enzyme of LTB_4_ catabolism. PPARα^−/−^ mice subjected to a topical application of 5-LOX-inducing inflammatory agent and LTB_4_ showed signs of tissue inflammation much longer (by about 30–40%) than wt mice, which were able to clear LTB_4_ from circulation much faster [80]. This experiment illustrates the importance of PPARα in the resolution of inflammation. This role of PPARα is necessary for regulation of the innate immune response, because proinflammatory lipid mediators, such as LTB_4_, are not only strong chemotactic agents for neutrophils and other leukocytes, but they also facilitate PMNs extravasation and diapedesis at the local site of inflammation and increase vascular permeability in this region [81,82]. By restricting LTB_4_ duration, PPARα alleviates three out of four inflammation symptoms (heat, flushing, and edema). Moreover, PMNs are not only recipients of LTB_4_ signals, but they are also activated to its production via a positive autocrine feedback loop [83]. Therefore, the PPARα-regulated LTB_4_ clearance protects from an overexaggerated inflammatory response and its transition from acute to destructive chronic state. The other eicosanoids, the products of either COX, i.e., prostaglandins PGD_1_, PGD_2_, PGA_1_, and PGA_2_, or 5-LOX product 8-(*S*)-HETE, also activate PPARα [84], which opens the possibility of modulating their impact on the cells with PPARα expression, whether in immunocompetent cells, such as monocytes/macrophages that express high levels of this receptor, or in the inflamed tissue. Such an activity contributes to tissue protection from inflammatory damage and facilitates regeneration.

### 4.4. PPARα Crosstalk with Pattern Recognition Receptors

Vertebrates take advantage of the PRR functions and employ them to sense all sorts of factors that induce tissue homeostatic imbalance. The PRR receptors are activated by the numerous compounds comprising specific structural entities referred to as the microbial-associated molecular patterns (MAMPs) or the Damage-associated molecular patterns (DAMPs). Several types of PRRs are broadly present in both immune and nonimmune cells, and their activation sparked by contacts with microorganisms, viruses, and some fragments of damaged cells or an alteration in the functioning of cell components (e.g., cytoskeleton or mitochondria malfunction or endoplasmic reticular stress) is the main trigger of the innate immunity response [85]. The PRRs can be divided into four main subfamilies: the Toll-like receptors (TLRs), the nucleotide-binding oligomerization domain (NOD)–leucin-rich repeat (LRR)-containing receptors (NLRs), the retinoic acid-inducible gene 1-like receptors (RLRs), and the C-type lectin receptors (CLRs) [11]. Nevertheless, some other cellular proteins can serve as PRRs in certain situations, e.g., the glycolytic enzyme, hexokinase II, which is able to spot the microbial sugar, *N*-acetylglucosamine, when this building block of peptidoglycan happens to be present in the cytoplasm [86]. In this section, we address the question of how PPARα may be involved in the MAMP and DAMP recognition process in various tissues and cells.

The noteworthy information on TLR and PPARα crosstalk comes from the studies on PPARα knockout (KO) mice and cells derived from these animals. The colonic macrophages from KO mice did not produce the regulatory IL-10, but secreted IL-6, IL-1β, and IL-12, potent inducers of Th1 and Th17 differentiation. Moreover, innate immune ILC3 cells isolated from the colon of PPARα KO mice produce lower levels of IL-22 compared with those from WT mice, which results in the impaired secretion of antimicrobial peptides and commensal dysbiosis. This indicates that PPARα regulates the ILC3 effector functions, which are important for both fighting infections and sustaining tolerance to commensal microbiota. The absence of PPARα affects the species composition of the microbiome and leads to increased representation of segmented filamentous bacteria (SFB). All these facts render the KO mice prone to gut inflammation development and are indirect proof of the critical role of PPARα activation in gut immunological homeostasis [30].

It is well known that interactions between the microbiota and intestinal cells engage Toll-like receptors [87], e.g., SFB regulate the process of Th17 differentiation in the intestine via activation of TLR5 by flagellin [88], and TLR4 ligand LPS from Gram-negative bacteria stimulates Th17 differentiation in vitro [89]. It seems that these events can be modulated by PPARα ligands. Accordingly, it was shown that macrophages from PPARα knockout mice are characterized by higher expression levels of mRNA for proinflammatory cytokines IL1β and IL6, as well as for COX-2 and NF-κB (p65) upon TLR4 ligand stimulation (LPS 50 ng/mL, 5 h), as compared to wild-type cells. It seems that PPARα deficiency speeds up LPS-induced inflammatory responses in murine macrophages [54]. Another study on PPARα KO mice indicated that PPARα was essential for the anti-inflammatory effect of acute exercises. Its absence induced overexpression of proinflammatory cytokines in LPS-treated macrophages isolated from mice 24 h post exercise [90].

TLR ligands can regulate PPARα activity, and PPARα agonists influence the expression of TLRs, as well as proteins involved in signaling from TLRs in various cells of both immune and nonimmune types. Becker et al. studied the involvement of LPS in the regulation of PPARα in murine lungs and showed that 24 h on from a prolonged LPS challenge (daily intranasal administration of 1 μg LPS for 4 consecutive days), a profound inhibition of PPARα mRNA expression took place [91]. LPS, peptidoglycan, and flagellin (ligands of TLR4, TLR1/2, and TLR5, respectively) strongly suppressed PPARα activity in rat astrocytes acting at the mRNA and protein expression level [92]. On the other hand, it was shown that fenofibrate, a pharmacological PPAR agonist, significantly inhibited the TLR4, MYD-88, and NF-κB mRNA expression, as well as TNFα production, in murine melanoma B16F10 LPS-stimulated cells [93]. The strong relationship between TLR4 and the PPARα signaling pathway was also clearly demonstrated in a model of endotoxin-induced uveitis. This study suggested that fenofibrate can also attenuate LPS-induced cytokine production, inhibit NF-κB signaling, and suppress TLR4 expression in retinal pigment epithelial cells. Simultaneously, LPS could act as a direct PPARα antagonist in a PPARα reporter cell line [94]. All these experimental data point to a subtle tuning and complicated interplay between activation of PPARα and the TLR signaling pathway, which is needed for the homeostatic balance between triggering and resolution of the inflammatory response in tissues.

### 4.5. PPARα and the Regulation of Inflammasomes

The inflammasomes, the complex molecular platforms formed in the cytoplasm (mainly in macrophages, but also in other nonimmune cells, such as endothelial and epithelial cells encountering various DAMPs and MAMPs), are now considered the key element of innate immunity. They are the multiprotein complexes composed of cytoplasmic sensors (mainly NLR family members), adaptive proteins (apoptosis-associated speck-like protein, ASC, or PY-CARD), and effectors (such as cysteine proteinase precursor or pro-caspase-1). In the case of some nonconventional inflammasomes, pro-caspase-1 is substituted by pro-caspase-11 in murine cells and pro-caspase 4/5 in human cells. The complex formation enables the proteolysis of pro-IL1β and pro-IL18 and the release of active cytokines into the cell microenvironment and bloodstream, which drives local or systemic inflammation [95]. Alternatively, the inflammasome formation induces a chain of events leading to pyroptosis—the special type of a programmed cell death connected to an inflammatory state. The molecular mechanisms contributing to inflammasome activity are not yet completely understood, but it is believed that the process of their formation requires two subsequent signals, e.g., LPS binding to TLR4 on the cell membrane as the primary signal and K^+^ efflux, cytosolic release of lysosomal cathepsins, or mitochondria-derived factors and reactive oxygen species generation as the secondary signal [96]. The regulation of inflammasome activation can occur at both signals on the post-transcriptional and post-translational levels [97].

It was shown in some animal models that PPARα activation can profoundly suppress the inflammasome-induced tissue injury, thereby contributing to the resolution of inflammation. This can be partially attributed to the downregulation of TLR expression by PPARα and interference with the primary step of inflammasome activation. However, in PPARα KO mice with lung inflammation caused by *Pseudomonas aeruginosa* introduction, a significant increase in expression of NLRP-3, ASC-1, and caspase-1, as compared with infected wt mice, was observed [98]. This indicates that PPARα expression background is also important for the supply of inflammasome building blocks.

Acute liver injury is a disease strongly connected with NLPR3 inflammasome activity. In the context of this pathology, Brocker et al. proposed a mechanism connecting fasting, PPARα, and the reduction in liver inflammation and injury. They showed that the long noncoding RNA gene Gm15441 contained a PPARα-binding site within its promoter, and the Gm15441 RNA expression was activated by PPARα ligand Wy-14643. Gm15441 suppressed its antisense transcript, encoding thioredoxin-interacting protein (TXNIP). This subsequently decreased TXNIP-stimulated NLRP3 inflammasome activation (Figure 2d) [99].

Moreover, it was shown that OEA, an endogenous bioactive lipid and a natural ligand of PPARα, prevented tissue damage in the onset of LPS/d-galactosamine (d-Gal)-induced acute liver injury. OEA administration increased PPARα expression in murine liver subjected to LPS/d-Gal treatment. In turn, the liver protein levels of IL-1β and NLRP3 inflammasome components, NLRP3 protein and pro-caspase-1, were enhanced after LPS/d-Gal injection in mice. The increase in these proteins was alleviated by OEA addition to the diet [100]. The OEA anti-inflammatory effects were also evident in dextran sulfate sodium (DSS)-induced mice colitis, and the effect was mediated by the inhibition of NLRP3, NF-κB, or MyD88-dependent pathways [101].

## 5. PPARα’s Role in the Innate Immunity Effector Processes: ROS/RNS Production

An important component of the innate immunity in animals is generation of active forms of oxygen (mainly superoxide) and active forms of nitrogen, mainly nitric oxide and its derivatives [102]. The form of nitric oxide synthase (NOS) traditionally associated with inflammation is the so-called inducible nitric oxide synthase (iNOS or NOS 2). NOS 2 belongs to the enzymatic family of nitric oxide synthases (NOS), being the evolutionarily most distant member of the family. NOS 2 may be expressed in numerous types of cells and tissues [103]. The other two, NOS 1 and NOS 3, also called ‘constitutive’ or Ca^2+^-dependent enzymes, are present constitutively in many tissues and cells of the organism, mainly but not solely in some neurons (NOS 1), as well as endothelial cells (NOS 3) [104]. They generate a lower level of NO than NOS 2, despite their comparable enzymatic activity in vitro [102]. Importantly, under various conditions, all NOS enzymes are a source of active forms of nitrogen and oxygen; in the absence of l-arginine, they simply produce superoxide and may be an important source of oxidative/nitrosative stress [105].

PPARα agonists may downregulate NOS 2 [106,107], while they stimulate both NOS 3 [108], which plays a protective role in the cardiovascular system, and NOS 1 (see [109,110]). NOS 2 is expressed de novo under the influence of proinflammatory factors [102], and, as it is not dependent on calcium, it can only be down regulated by inhibition of the enzymatic activity or proteolytic degradation of the enzyme. NOS activity also depends on competition with the alternate substrate consumer arginase, which produces urea and l-ornithine instead of l-citrulline and nitric oxide [111,112]. The possibility of switching the main path of l-arginine metabolism from the generation of NO and citrulline to the generation of urea and ornithine is a basis for the functional diversification of M1 and M2 macrophages. M1 macrophages, unlike M2 macrophages, generate free radicals and are the proinflammatory type of these cells (as mentioned in Section 3). They contribute to the development of inflammation-driven tumors [107]. PPARα, as an attenuator of inflammation and free-radical production, acts in this case as an antitumor agent. Parallel to tumor progression and diversification of the tumor macrophageal phenotype toward M2, the situation becomes more ambiguous and unpredictable. The actual effect of activation of PPARα clearly depends on the type of tumor and its phase of development [108]. Indeed, fenofibrate inhibited the development of micrometastases of melanoma BHM in Syrian hamster lung, but did not affect the kinetics of the primary tumor growth, nor the progression of macro-metastases [113]. It must be added that, recently, particular attention has been paid to the possibility of manipulation of NOS 2 activity by its selective inhibitors in order to achieve a desirable level of human monocyte physiological response [114].

The second mechanism of innate defense that involves the production of highly reactive small chemical molecules is respiratory (or oxidative) burst carried out by phagocytes. PPARα agonists were shown to increase macrophage microbicidal activity through intensification of ROS production during respiratory burst. This was caused by PPARα-dependent elevated expression of crucial transmembrane (gp91phox) and cytosolic (p47phox and p67phox) components of NADPH oxidase [115]. Interestingly, increased ROS production led to the generation of oxidized low-density lipoproteins (oxLDL), which further stimulated PPARα activation. Activated PPARα downregulated NO production via transrepression of iNOS [115]. This is an example of PPARα differently regulating various innate immunity effector molecules, in this case, ROS and RNS. An unexpectedly interesting transcriptional regulation occurs in the promoter of another gene crucial for the generation of reactive species during respiratory burst, namely, myeloperoxidase (MPO). The human promoter of this gene contains primate-specific Alu elements that are repetitive DNA mobile fragments spread throughout the human genome in about 1 million copies [116]. The Alu fragment in the MPO gene promoter contains four hexamer sequences identical to or closely resembling canonical PPAR response elements (PPREs): AGGTCA, with 2 or 4 bp spacing between them [117]. The third and fourth hexamers serve as PPREs and accommodate PPARα/RXR or PPARγ/RXR heterodimers, which enables transcriptional regulation by PPAR ligands. Surprisingly, MPO expression is regulated by PPARα agonist GW9578 and PPARγ agonist MCC-555 in opposite directions in human macrophages, depending on the differentiation pathway; MPO is significantly downregulated in macrophages derived from MG-CSF-treated monocytes and upregulated in M-CSF differentiated cells [117]. The difference could probably be attributed to the differential utilization of nuclear co-repressors, such as NCoR or silencing mediator of retinoid and thyroid receptors (SMRT), in macrophages differentiated with GM- vs. M-DAMP [117]. Notably, such a mode of regulation is entirely human-specific, because mice do not possess Alu elements in their genome.

## 6. PPARα as an Immunomodulator during Infections

Truly immunomodulatory action does not lie in the unilateral inhibition or activation of all inflammatory processes, but in selective influence on the chosen aspects of innate immunity. Such an immunomodulatory action of PPARα has been observed in parasitic or microbial infections. One example of such an activity relates to the induction of M2 polarization in macrophages of patients infected with *Trypanosoma cruzi*, a parasitic euglenoid, which is responsible for Chagas disease development. The experiment carried out on the infected mice showed that PPARα agonist Wy-14643 elevated the expression of M2 macrophage markers, arginase-1, mannose receptor (CD206), Ym1, and TGFβ, and decreased the production of proinflammatory molecules characteristic of the M1 phenotype, such as iNOS, NO, IL-1β, IL-6 and TNFα [118]. However, this phenotypic switch was accompanied by a PPARα (but not PPARγ)-dependent increase in phagocytic capacity and efficiency of parasite phagocytosis [118]. These results indicate that PPARα activation might have therapeutic significance, because its immunomodulatory action, on the one hand, strengthens macrophage effector capacity, but, on the other hand, helps to alleviate severe chronic inflammation associated with Chagas disease, which is destructive to various organs.

Similar immunomodulatory activity of PPARα in the context of phagocytosis was described in primary peritoneal macrophage and microglia cultures treated with several PPARα agonists: endogenous cannabinomimetic (see below), PEA, fenofibrate, or palmitic acid [119]. These compounds, particularly PEA, significantly enhanced phagocytosis and intracellular killing of *E. coli* by macrophages and microglial cells. Although PEA pretreatment reduced the levels of proinflammatory cytokines (IL-1β, IL-6, and TNFα) and chemokines (CXCL1) in the tissues of mice subjected to intracerebellar or intraperitoneal *E. coli* infection, it induced a very effective bacterial clearance from blood, spleens, and cerebelli, which translated into improved survival of these animals [119]. These results suggest a prophylactic potential of PPARα activation in the case of bacterial infections.

Another example illustrating that the exaggerated inflammatory response is not beneficial for the host is tuberculosis infection. In this case, PPARα’s immunomodulatory and metabolic roles are connected, leading to a better outcome for wt mice infected with mycobacteria (*Bacillus* Calmette–Guerin or *M. tuberculosis*) in comparison with PPARα KO mice [120]. The absence of PPARα resulted in more rapidly increasing intracellular bacterial load in macrophages, heavier bacteremia in the lungs, spleen, and liver, and a significantly higher level of inflammatory cytokines TNFα and IL-6 in the lungs, as compared to wt PPARα mice. The exaggerated inflammatory response was associated with a higher number of granuloma lesions in the lungs of PPARα KO mice. Granuloma lesions are the manifestation of unsuccessful host defense against mycobacteria, because they are full of dead leukocytes, damaged lung tissue multinucleated giant cells, and macrophages converted to foam cells, filled with lipid-containing vesicles, which create a favorable energy source for surviving and proliferating mycobacteria [121]. Pharmacological PPARα agonists GW7647 and Wy-14643 induced phagosomal maturation through activation of transcription factor EB (TFEB) and significantly reduced the survival of intracellular bacteria, which resulted from increased fatty-acid β-oxidation and elimination of lipid-rich bodies [120]. This is an example of the interconnection between PPARα-mediated lipid catabolism and its immunomodulating effects, which support effective antimicrobial innate defense.

Despite a large body of evidence documenting the beneficial outcomes of PPARα activation in various diseases with an inflammatory background, there are also certain conditions in which PPARα-mediated immunomodulation is hazardous. The illustrative example is a situation where, after viral influenza infection, a subsequent bacterial (e.g., staphylococcal) superinfection occurs. Antibiotic-resistant *Staphylococci* are frequent cause of life-threatening nosocomial infections in patients hospitalized due to viral pulmonary infections. Tam and colleagues [122] found out that the presence of PPARα was responsible for a more severe course of superinfection and a higher mortality in wt mice as compared to PPARα KO mice. Viral infection that was induced prior to challenge with *S. aureus* led to increased PPARα expression in lungs. Moreover, the lipidomic analysis of bronchoalveolar lavage fluid from infected mice revealed that superinfection resulted in a significant enrichment of several inflammatory lipid mediators, such as LOX product LTE_4_ and CYP450 products 11,12-dihydroxyeicosatrienoic acid (11,12-diHETrE) and 14,15-diHETrE, as compared to single infection, whether viral or bacterial. 14,15-diHETre is a very potent PPARα agonist [123]. The inhibition of NF-κB signaling mediated by activated PPARα led to a blunted proinflammatory response to bacteria and loss of control over bacterial growth, which inflicted higher mortality [122]. Superinfection caused the decreased expression of macrophage inflammatory genes IL-1β, IL-6, CXCL5, and MMP-9, as well as a scavenger receptor Marco, which resulted in less efficient phagocytosis and heavier bacterial burden. Moreover, PPARα activation led to increased necroptosis (a programmed RIPK3 kinase-dependent lytic cell death), which was responsible for lung tissue damage and dramatically worsened the condition of infected animals [122].

The still scarce, but gradually emerging experimental data indicate that PPARα affects the innate host response to viral infections. Such an involvement is beneficial in certain situations, but could be detrimental in other conditions. The overexpression of PPARα homolog in a grouper fish (*Epinephelus coioides,* EcPPARα) blocked interferon- and NF-κB-induced cytokine expression during viral infections, which led to acute cytopathic injuries and heavier multiplicity of infection [124]. The topic of viral infection onset is currently very important due to its relationship with the ongoing COVID-19 pandemic. A study performed on primary human bronchial epithelial cells infected with SARS-CoV-2 revealed severe alterations in the gene transcription pattern that manifested endoplasmic reticular and mitochondrial stress, metabolic reprogramming toward intensive lipid synthesis and accumulation, impaired fatty-acid oxidation, and upregulated aerobic glycolysis via activation of the NF-κB pathway [125]. Such a metabolic signature suggests that infection impairs PPARα signaling. Therefore, the restoration of PPARα activity could be beneficial through reversal of these changes and metabolic ‘repair’. Indeed, the treatment of the infected cell cultures with PPARα ligand fenofibrate alleviated the dysregulation of lipid metabolism, blocked infection-induced phospholipid accumulation, and remarkably decreased viral load by 100-fold within 3 days and 1000-fold within 5 days [125]. These results seem to support the hypothesis that fenofibrate treatment could alleviate the acute infection symptoms during COVID-19 by supporting fatty-acid metabolism in alveolar epithelial cells, improving pulmonary endothelial cell function, and calming down the cytokine storm, leading to a better outcome for the patients [126].

## 7. Interplay between PPARα and the Endocannabinoid System: Implications for Inflamma-Tion, Neuroprotection, and Analgesia

### 7.1. Analgesic Lipid Mediators as PPARα Agonists

Mechanical tissue damage, hypersensitivity reactions or local infection result in inflammation, which evokes a nociceptive response and pain. Pain signals are elicited by proalgesic lipid mediators, such as lysophospholipids and PDE_2_, or hydroxylated derivatives of linoleic acid (e.g., 13-hydroxyoctadecanoic acid, 13-HODE), which increase the excitability of nociceptive neurons [127]. Nevertheless, another group of endogenous lipid mediators possesses opposite, analgesic activity. Acting through cannabinoid receptors CB1 and/or CB2, they mitigate the excitability of sensory nociceptive neurons. This is a part of the so-called endocannabinoid system, which includes the ligands *N*-arachidonoylethanolamine (AEA, anandamide) and 2-arachidonoyl-glycerol (2-AG), which were first discovered, and their receptors, cannabinoid receptors CB1 and CB2 expressed in the CNS and immunocompetent cells, respectively, as well as TRPV1 and endocannabinoid-synthesizing and -degrading enzymes [128,129]. Later, other fatty-acid ethanolamides (FAEs), such as *N*-palmitoylethanolamide (PEA) and *N*-oleoylethanolamide (OEA), were detected in mammalian and invertebrate tissues [130,131,132]. OEA and PEA are biologically relevant and potent PPARα agonists, with EC_50_ values of 0.12 μM and 3 μM, respectively [44,133], which links PPARα with the endocannabinoid system. Numerous biological hormone-like functions of OEA and PEA are widely known, including analgesic and anti-nociceptive cannabinomimetic activities, although they are not bona fide CB1 or CB2 agonists [134]. Endocannabinoids and cannabinomimetics are synthesized on demand from membrane phospholipids, but can also be accumulated intracellularly in lipid droplets [135,136]. They are abundantly present in the brain, leukocytes, gastrointestinal tract, and other tissues [137,138,139].

The most common FAE biosynthesis route involves the formation of *N*-acyl-phosphatidylethanolamine from phosphatidylethanolamine by calcium-dependent *N*-acyl-transferase and subsequent conversion to *N*-acyl-ethanolamine by *N*-acyl-phosphatidylethanolamine-hydrolyzing phospholipase D (NAPE-PLD) [140]. Several other biosynthesis pathways that engage other phospholipases and glycerophosphodiesterases are also possible (for a review, see [128]). Endocannabinoids are absorbed by cells and metabolized by intracellular fatty-acid amide hydrolase (FAAH) or *N*-acylethanolamine-hydrolyzing acid amidase (NAAA) [141].

**Figure 3:**
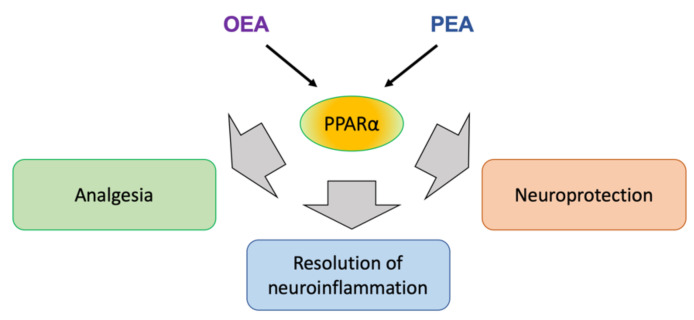
Endocannabinoids OEA and PEA exert analgesic, anti-inflammatory, and neuroprotective actions through PPARα activation. A detailed explanation is provided in the text.

OEA and PEA exert analgesia and reduce nociception in various animal models of inflammatory pain [142,143]. PEA and synthetic PPARα ligands (GW7647, Wy-14634, perfluorooctanoic acid) produce analgesic effects and strongly reduce edema in chemically induced models of inflammation [142,144,145,146]. Although, in some cases, OEA acted independently of PPARα presence [143], PEA-induced nociception and anti-inflammatory actions were exerted through PPARα [142,145]. Importantly, PEA-mediated activation of PPARα in CNS through intracerebroventricular PEA application was able to reduce peripheral inflammatory response (a paw edema after carrageenan injection) [146]. This demonstrated a distant endocrine action of PEA, despite the molecular mechanism involving inhibition of the NF-κB signaling pathway in CNS tissue [146]. A PPARα involvement was also demonstrated in the experiments with a synthetic PPARα agonist GW7647, which induced synergistic enhancement of AEA analgesic properties in a chemically induced inflammatory pain model [145,147]. The antinociceptive action of GW7647 depended on the activity of large conductance potassium channels, which further supported an involvement of endocannabinoid system [145,147]. The potentiation of endocannabinoid binding to CB1 and CB2 receptors by cognate molecules, which are not agonists themselves, was observed and named ‘the entourage effect’ [148]. In the case of AEA, PEA, and OEA, such an effect could be explained by FAAH engagement in PEA and OEA hydrolysis, sparing the large pool of AEA from degradation and allowing it to activate CB receptors. Indeed, the entourage effect has been described as an enhanced vasodilation activity of AEA through TRPV1 by PEA and OEA in the endothelium [149]. In summary, all these results indicate that PPARα signaling contributes to inflammatory pain control through cannabinomimetics OEA and PEA (Figure 3) [127].

### 7.2. PPARα Involvement in Resolution of Neuroinflammation

The presence of OEA and PEA in CNS implicates their activity in the physiology of neurons and glial cells. Both compounds were shown to exert beneficial effects by counteracting the glial inflammatory responses and by providing cytoprotection over neuronal cells and their activities in various neuropathic states. Neuroinflammation and exaggerated glial reactivity are associated with numerous neurodegenerative diseases, traumatic injuries, ischemia/reperfusion stress, and neuropathic pain [150,151,152]. The brain is regarded as ‘an immune-privileged’ organ, protected from peripheral proinflammatory stimuli by the blood–brain barrier, but microglia, astrocytes, and mast cells are capable of triggering neuroinflammation [153]. Aberrant or chronic activation of these cells in the CNS leads to increased expression of TLRs, cytokines (TNFα, IL-6), chemokines (CXCL6) metalloproteinases, ROS, and RNS, which results in the loss of calcium homeostasis, neuronal damage, or apoptosis [151,152,153]. The potential of lipid amides, called ALIAmides (autacoid local injury antagonists) to counteract neurogenic inflammation and mast-cell degranulation, was proposed by Rita Levi-Montalcini, a Nobel laureate (1988), for her discoveries in the field of neurobiology [154]. Indeed, numerous studies demonstrated that OEA and PEA, classified as ALIAmides, could provide neuroprotection via downregulation of inflammatory responses in the brain through modulation of glial cell functions. Benito and colleagues discovered that *N*-fatty acylethanolamines (OEA, PEA, AEA) and synthetic agonists of PPARα (Wy-14643) and PPARγ (troglitazone) alleviate the inflammatory response induced by the treatment of astrocytes with β-amyloid peptide fragments [155]. The anti-inflammatory effects were mediated by PPARα, PPARγ, and TRPV1 activity, but not through CB1 or CB2 [155]. The neuroprotective action of PEA and an endocannabinoid 2-AG was observed in an excitatory model of neuronal damage in organotypic hippocampal slice cultures [156]. PEA and 2-AG rescued about 50% of neurons from NMDA-induced cell death, acting on microglial cells, albeit through different and mutually suppressing mechanisms. PEA blocked microglial inflammatory activities, such as NO production and the acquisition of ameboid morphology, characteristic of an activated condition [156]. These effects were associated with PPARα nuclear translocation, which suggests its involvement in the process.

### 7.3. PPARα-Mediated Regulation of Microglia and Macrophage Functions

The glia-directed activity of PEA was studied by Scuderi and coauthors, who, in a series of papers, demonstrated that PEA or synthetic PPARα agonists, in a PPARα-dependent manner, decreased markers of glial inflammation and improved neuronal viability in animal models of Alzheimer’s disease, as well as in mixed glio-neuronal cell cultures and organotypic neural cultures [157,158,159]. The immunomodulatory activity of PEA and the interplay between PPARα and the endocannabinoid system were also analyzed in primary microglial and macrophage cultures [160]. This study revealed that CB2 mRNA and protein levels were significantly increased by the treatment with PEA and a synthetic PPARα agonist GW7647, and this effect was evoked by the PPARα/RXR heterodimer binding to the promoter and transactivation of the gene encoding CB2 [160]. PEA induced microglial effector functions in a PPARα-dependent manner and improved the phagocytosis and killing of *Porphyromonas gingivalis* by microglia and chemotaxis to 2-AG [160]. In addition to the modulation of antimicrobial phagocytosis-based defense, PEA can modulate regenerative functions of macrophages, such as efferocytosis (i.e., phagocytosis and clearance of apoptotic cells) [161]. PEA is produced endogenously by M2c-polarized but not M1-polarized macrophages [161]. Exogenous chronic administration of PEA limited early plaque formation, protected from accumulation of the proinflammatory M1 macrophage within the plaque, and promoted efferocytosis by M2a- and M2c-polarized macrophages, which delayed the onset of arteriosclerosis [161]. These results show that endogenous PPARα ligand PEA is capable of modulating microglia and macrophage biological functions.

### 7.4. PPARα’s Role in Restoration of Neural Function after Injury or Infection

Neuroprotective OEA activity was also demonstrated as an inhibition of so-called glial scar (i.e., zones enriched with reactive inflammatory astrocytes, microglia, fibroblasts, and accumulated extracellular matrix components) formation, after focal cerebral ischemia injury [162]. Glial scar is a natural physiological reaction to injury, but it impedes neurite formation, axon regrowth, and recovery after brain stroke. OEA increased PPARα expression in the cerebral cortex and downregulated glial scar markers (S100B, glial fibrillary acidic protein GFAP, metalloproteinases MMP-2, MMP-9, and neurocan) in the ischemic region through a PPARα-dependent mechanism [162]. Importantly, these biological processes translated into a better recovery of motor function in mice after stroke [162]. OEA also decreases the inflammatory response of endothelial cells (such as IL-6, IL-8, ICAM-1, and VCAM expression) evoked by TNFα, in a PPARα- and CB2-dependent manner [163].

The biological activities of OEA and PEA seem similar and sometimes overlap, but are not always identical, as shown in different experimental settings. An intriguing difference between OEA and PEA actions was observed in a study that analyzed functional impairments of neurological functions in an animal model of neonatal anoxia/ischemia-induced brain injury [164]. PEA, but not OEA treatment was capable of limiting hippocampal astrogliosis markers (e.g., ionized calcium-binding adaptor protein Iba-1, GFAP) and restoring PPARα protein expression in anoxia/ischemia-affected brain regions [164]. These effects were associated with improved cognitive abilities and a better recovery of spatial and recognition memory, as compared to control animals subjected to anoxia/ischemia [164]. Nevertheless, OEA was proved effective in ameliorating cognitive deficits and in supporting neurogenesis in ischemia-affected brain regions of rats subjected to middle cerebral artery occlusion [165].

An important immunomodulatory action of OEA and PEA involves TLR3 signaling during the innate response to viral infections. A recent report by Flannery et al. [166] demonstrated that intracerebroventricular administration of a TLR3 ligand, viral mimetic polyinosinic–polycytidynic acid (poly I:C), led to the induction of hypothalamic interferon- and NF-κB-regulated pathways of proinflammatory gene expression and hyperthermia. The treatment with both OEA and PEA attenuated TLR3-mediated hyperthermia, but only OEA (not PEA) was effective in the downregulation of poly I:C-induced inflammatory gene expression, including TNFα, iNOS, IL-1β, COX-2, interferon gamma-induced protein 10 (IP-10), and interferon-regulated factor IRF7. The fact that the PPARα antagonist GW6471 attenuated these effects indicated the PPARα involvement in this regulation [166]. These results have important implications for the current pandemic of SARS-CoV-2 infections, which often cause complications within the CNS, manifested by neurological and mental disorders, such as impaired memory, attention, anxiety, depression, and dementia [167].

### 7.5. PPARα and Endocannabinoid Involvement in the Regulation of Mast-Cell Functions

Mast cells are important innate immunity cells that, due to their rapid degranulation, can control the onset of inflammation in various tissues. PEA was shown to reduce local accumulation and the activation of mast cells in various inflammatory models: (i) after substance P injection to ear pinna [154], (ii) during chemically induced allergic dermatitis in mice [168], (iii) in myelin basic protein (MBP)-induced neuronal injury in a neuron–glia–mast cell coculture model of multiple sclerosis [169], (iv) in rat mast cell line RBL-2H3 [170], (v) after ischemia/reperfusion inflammatory injury of intestine after splanchnic artery occlusion in mice [171], and (vi) during chemically induced colitis which serves as an animal model of inflammatory bowel disease [172]. In all these experimental models, PEA suppressed a variety of effector reactions produced by mast cells or other leukocytes, such as chemotaxis, degranulation, enzyme release, and induction of proinflammatory cytokines. This suppression of mast-cell activity led to alleviation of inflammatory tissue damage and improved physiological tissue function. A common molecular mechanism could be involved in these effects, because, regardless of the model used, they were mediated, at least partially, by PPARα and CB2 activation [168,169,170], as well as, in some cases, by GPR55 and TRPV1 [172], which further supports the role of PPARα in the modulation of innate immunity and its connections with the endocannabinoid system.

However, a very intriguing recent discovery has shed new light on the connection among cannobinomimetics, mast cells, and metabolism, namely, ketogenesis. The publication from Daniele Piomelli’s group revealed the unexpected role of histamine secreted by mast cells as a mediator necessary to induce ketogenesis in the liver in the state of food deprivation [173]. The mode of metabolic regulation involves an OEA-mediated action on hepatocytes. Routinely, after feeding, OEA is produced in the small intestine from consumed dietary lipids and takes part in food intake control as a satiety mediator via PPARα activation [133,174]. However, during food deprivation, ketogenesis depends on liver-derived OEA. A crucial role in this process is played by a population of mast cells that reside in the gastrointestinal tract and release histamine in the fasting state. Histamine enters the liver through portal circulation and stimulates hepatocytes to OEA secretion via activation of histamine H1 receptors [173]. Furthermore, OEA binding to PPARα in hepatocytes activates transcription of PPARα-target genes that control ketogenesis, including ACAT1, HMGSC2, and Fgf21 [173]. These results provide a novel link between mast cells as innate immunity effectors, cannabinomimetic PPARα ligand OEA, and PPARα-dependent ketogenesis as a metabolic response to fasting.

## 8. Evolutionary Aspects of PPARα-Mediated Immunomodulation

One of the crucially important features of the innate response is the speed and immediateness of the reaction to menacing invaders. In higher vertebrates, the accurate and prompt launching of the innate mechanisms buys time for the preparation of systemic adaptive immunity. In invertebrates, the effectiveness of innate immunity is a matter of life and death. The precise regulation of the innate responses is a multithreaded process that engages various signaling pathways, including the activity of nuclear receptors, such as PPARs. Such a regulation determines the success in coping with parasitic, viral, and bacterial infections, in addition to providing a hospitable environment for commensal microbiota and restricting inflammation-related tissue damage and injury.

PPARs and NOS serve as an illustrative example of how the elements of innate immunity and their regulatory mechanisms coevolved in the animal kingdom. On the one hand, NOS belongs to a large family of evolutionarily ancient enzymes that includes numerous pro- and eukaryotic flavodoxins [175,176]. There have been several hypotheses of their reciprocal relationship in invertebrates in the function of hemolymph homeostasis maintenance and the destruction of pathogens, i.e., probably unified in hemocytic NOS, as is the case for horseshoe crabs [175,177]. On the other hand, PPARs, despite their origin in the nuclear receptor family that emerged in metazoans, evolved in animals only as late as in the branch of Deuterostomata, whereas, in chordates, their presence dates from the evolution of Branchiostomata [178]. Consequently, they are present in all the vertebrates, but (except for Branchiostomata) absent in invertebrates [178]. Their presence seems to correspond to the evolution of the immune system and adipose tissue, but their tissue specificity does not overlap with their functional diversification. The most basic branch of this family seems to be represented by PPARγ, and the evolution of the whole family comprised two duplications of the genes, the first moving PPARγ apart, and the other dividing the other group into the PPARβ and α subfamilies [179]. This must have taken place on the level of ancient, primitive Teleostei [178,179].

**Figure 4:**
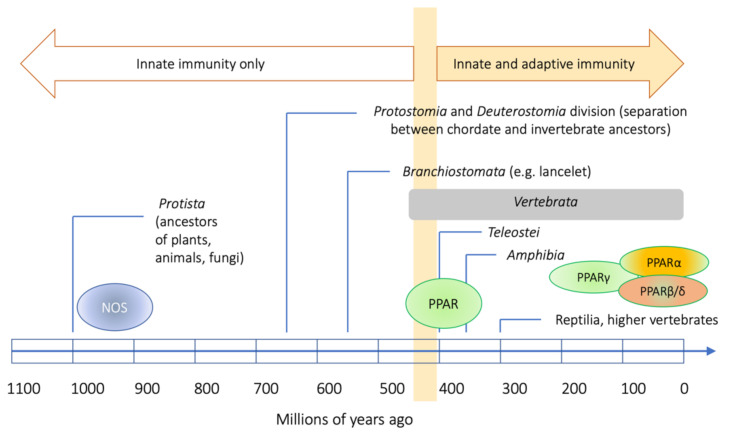
Long evolution of NOS as a background for shorter vertebrate-related evolution of PPARs and its involvement in the immune response in various animal phyla. The time scale is only illustrative and was based on [181].

Meanwhile, the diversified NOS family tree must root as deeply as in some Protista, as present in a differentiated side-branch in slime molds, fungi, and practically all Eukaryota including (a loosely related variant) high plants (*Arabidopsis thaliana* [180]). This may explain the engagement of PPARs in the functioning of various NOS in vertebrates. Upon evolution, the diversification of the NOS family has been consistently appreciated, whereas the engagement of PPARs in various aspects of NOS functioning may have been more or less accidental (Figure 4).

## 9. Conclusions and Perspectives

PPARα as a transcription factor exerts a strong impact on cellular metabolism and intracellular signal transduction events, which alters the physiology and behavior of PPARα-expressing cells of both immune and nonimmune provenance. These physiological alterations underlie the immunomodulatory actions of PPARα presented in previous chapters. The broad spectrum of actions of endogenous and pharmacological PPARα agonists directed toward the immune system encourage the development of more commonly used therapeutic application of PPARα-targeted solutions in various infectious diseases and disorders of immunological background. The currently ongoing SARS-CoV-2 pandemic has created a dire need to revise the canonical approaches to the treatment of viral infections and has opened an unexpected possibility for new attempts, such as applying PPARα agonists to calm down the destructive cytokine storm in severe COVID-19 cases.

## Figures and Tables

**Table 1:** Chemical structures of PPARα endogenous agonists, synthetic agonists used in experimental studies, clinically used pharmacological agonists, and synthetic antagonists, including examples of novel *N*-phenylsulfonylamide compounds (the structures of 3- and 10- series according to [64]).

	PPARα Agonists and Antagonists
Natural agonists	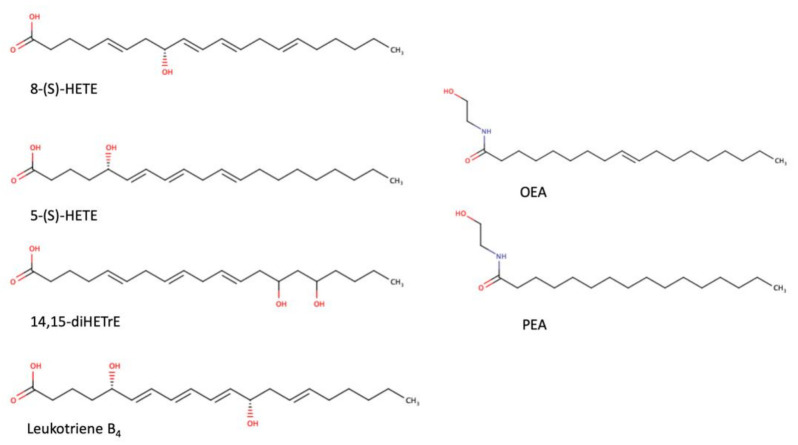
Synthetic agonists	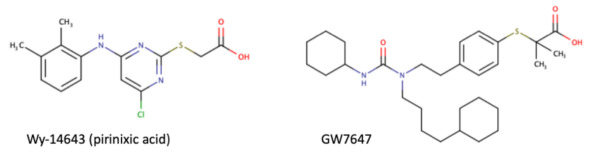
Agonists applied in clinic: fibrate derivatives	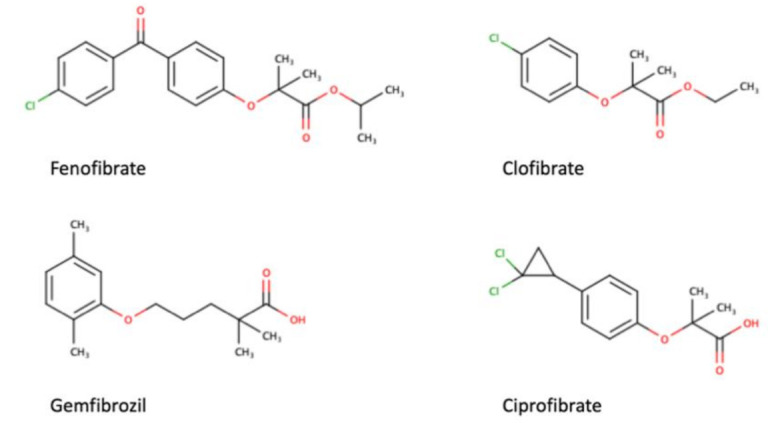
Synthetic antagonists	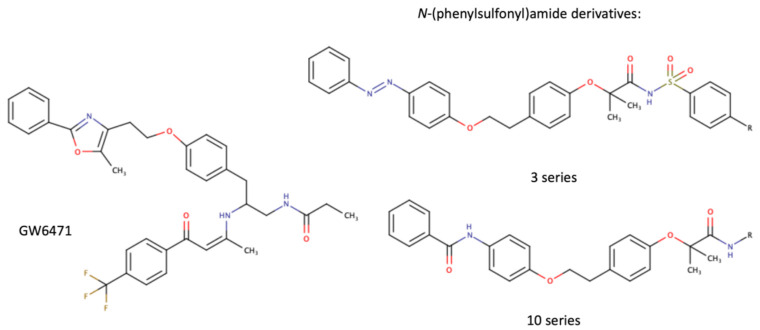

## Abbreviations

2-AG, 2-arachidonoyl-glycerol; ACAT1, acetoacetyl-CoA thiolase 1; AEA, *N*-arachidonoylethanolamine; AMPs, antimicrobial peptides; AP-1, activation protein 1; CB, cannabinoid receptors; CLRs, C-type lectin receptors; COX, cyclooxygenase; CSF, colony-stimulating factor; DAMPs, damage-associated molecular patterns; DOPA, di-hydroxyphenylalanine; FAAH, atty-acid amide hydrolase; FAEs, fatty-acid ethanolamides; FAO, fatty-acid oxidation; FGF21, fibroblast growth factor 21; FREPs, fibrinogen-related proteins; HETE, hydroxyeicoatetraenoic acid; HMGCS2, 3-hydroxy-3-methylglytaryl-CoA synthetase 2; HPETE, hydroperoxyeicosatetraenoic acid; IDO, indoleamine-2,3-dioxygenase; IL, interleukin; ILCs, innate lymphoid cells; IRF, interferon-regulated factor; JAK, Janus-activated kinase; JNK, c-Jun N-terminal kinase; KO, knockout; LOX, lipoxygenase; LPS, lipopolysaccharide; LT, leukotriene; MAMPs, microbial-associated molecular patterns; MBP, myelin basic protein; MCP1, monocyte chemoattractant protein 1; MDSCs, myeloid-derived suppressor cells; MMP-9, matrix metalloproteinase 9; NAAA, *N*-acylethanolamine-hydrolyzing acid amidase; NAPE-PLD, *N*-acyl-phosphatidylethanolamine-hydrolyzing phospholipase D; NCoR, nuclear receptor co-repressor; NF-κB, nuclear factor κB; NLR, nucleotide-binding oligomerization domain (NOD)–leucin-rich repeat (LRR)-containing receptors; NO, nitric oxide; NOD, nucleotide-binding oligomerization domain; NOS, nitric oxide synthase; OEA, oleylethanolamide; PAMPs, pathogen-associated molecular patterns; PEA, palmitoylethanolamide; PG, prostaglandin; PPAR, peroxisome proliferator-activated receptor; PPRE, peroxisome proliferator response element; PRRs, pattern-recognition receptors; RIG1, retinoic acid inducible gene 1; RLR, retinoic acid inducible gene 1 (RIG1)-like receptors; RNS, reactive nitrogen species; ROR, retinoid orphan receptor; ROS, reactive oxygen species; RXR, retinoid X receptor; SAPK, stress-activated protein kinase; SMRT, silencing mediator of retinoid and thyroid receptors; STAT, signal transducer and activator of transcription; TF, tissue factor; TFEB, transcription factor EB; TGF, transforming growth factor; TLR, Toll-like receptors; TNF, tumor necrosis factor; TRPV1, transient receptor potential cation channel vanilloid subfamily member 1; TXNIP, thioredoxin-interacting protein.
